# Hypertension and Chronic Kidney Disease: A Meta-Analysis

**DOI:** 10.7759/cureus.113669

**Published:** 2026-07-30

**Authors:** Saad N Almutairi, Ali H Alarbash, Abdullah Almutairi, Hamad R Alazmi, Abdulrahman Almutairi, Mariam A Alkandari, Ahmad Abdalla, Ahmad Alzufairi

**Affiliations:** 1 Internal Medicine, Al Jahra Hospital, Al Jahra, KWT; 2 Pediatrics, Mubarak Al-Kabeer Hospital, Jabriya, KWT; 3 Internal Medicine, King Faisal Specialist Hospital and Research Center, Madinah, SAU; 4 Internal Medicine, Farwaniya Hospital, Sabah Al-Nasser, KWT; 5 Internal Medicine, Makkah Al-Mukarramah Health Cluster, Mecca, SAU; 6 General Surgery, Farwaniya Hospital, Sabah Al-Nasser, KWT

**Keywords:** chronic kidney disease, hypertension, meta analysis, renal impairment, risk factors, systematic review

## Abstract

Hypertension (HTN) is considered one of the global health concerns and is a significant risk factor for chronic kidney disease (CKD), affecting millions worldwide. Meta-analysis is a reliable statistical method for integrating empirical results selected from various studies to formulate evidence-based conclusions. This meta-analysis was performed to evaluate the impact of HTN on developing CKD across various settings and demographics. Several electronic databases, including PubMed and Google Scholar, were searched to collect relevant research papers published between January 2015 and September 2024. The inclusion criteria were determined by applying the PICOS principle (patients, intervention, comparison, outcomes, and study design). The primary factors studied were renal failure, male gender, and smoking, according to the research findings. Data were extracted from 13 case-control studies that investigated 17 risk factors, and effect sizes were calculated utilizing a random-effects model to address any heterogeneity. Statistical analyses were performed using Comprehensive Meta-Analysis V4 software, employing a random-effects model to assess odds ratios (OR) and 95% confidence intervals (CI), along with heterogeneity assessment via Cochran’s Q test. The meta-analysis revealed a significant association between HTN and CKD, with renal impairment showing the strongest correlation (odds ratio (OR)=3.846, 95% confidence interval (CI): 2.817-5.251, p<0.001). Additional risk factors included male gender (OR=1.701, 95% CI: 1.082-2.674, p<0.001) and smoking (OR=1.732, 95% CI: 1.208-2.482, p<0.001). The results showed geographical differences in risk factors, emphasizing the impact of environmental and lifestyle factors on disease prevalence. This meta-analysis establishes a robust association between HTN and CKD, highlighting renal impairment, male sex, and tobacco use as significant risk factors. The findings highlight the necessity for focused treatments, such as improved HTN management, lifestyle changes, and early screening, especially in resource-constrained environments. Future research should concentrate on region-specific solutions and enhancing CKD diagnosis methodologies to alleviate the global burden of HTN-related kidney disease.

## Introduction and background

Hypertension (HTN), sometimes referred to as the silent killer, is one of the most common diseases that is considered a risk factor for several health problems such as chronic kidney disease (CKD), heart attacks, strokes, and other serious health problems [1]. HTN is caused when the force of the blood against the artery walls is high enough for a long time period, causing health problems [2]. HTN is an escalating issue in global health, as it is defined by European guidelines as systolic blood pressure (SBP) in the office (≥140 mmHg) and/or diastolic blood pressure (DBP) of ≥90 mmHg [3].

In 2019, an estimated 1.28 billion adults aged 30-79 years worldwide had hypertension, and blood pressure was controlled in fewer than one in four women and one in five men with hypertension [4]. The WHO considers HTN as a significant contributor to premature death globally. The consequences of HTN extended beyond adulthood; higher blood pressure during childhood predicts future hypertension, emphasizing the importance of early identification and intervention [5].

The kidneys, which filter waste from the blood, are especially affected by chronic HTN. This damage can affect the delicate filtering units of the kidneys by the exerting high pressure, known as nephrons, which can decrease renal function and initiate a gradual decline in renal function [6]. The damage to kidneys is often irreversible and in advanced cases it may result in end-stage renal disease (ESRD), requiring dialysis or a kidney transplant for survival. CKD is also considered a worldwide health issue, with an estimated prevalence of 11%-13%, with 80%-85% of these patients suffering from hypertension [7]. According to studies, there is a bidirectional relationship between HTN and CKD. As the body's capacity to regulate blood pressure weakens as kidney function decreases, it can result in increasing blood pressure levels. The progression of both conditions is accelerated by this vicious cycle [8].

Furthermore, the burden of CKD and HTN is especially severe in populations with limited access to healthcare resources. There are no early symptoms for CKD patients until they reach the later stages of the disease, which requires early detection and effective blood pressure management to prevent severe consequences. Various studies showed that there are also other risk factors leading to the increasing risk of kidney damage, such as diabetes, obesity, and a family history of kidney disease [9]. This complex relationship between HTN and kidney damage demonstrates the significance of having a deeper understanding of the risk's magnitude and the methods in which optimal blood pressure control may mitigate it.

Methods

Eligibility Criteria

Published related articles from January 2015 until September 2024 were included. We selected 2015 as the start year for the search to provide recent and uptodate meta analysis considering the subject of the research. We included all studies (without language restrictions) that reported CKD (based on clinical assessment) as a result of hypertension in adults. Hypertension was defined as systolic BP (SBP) ≥140 mmHg and/or diastolic BP (DBP) ≥90 mmHg [10].

**Figure 1:**
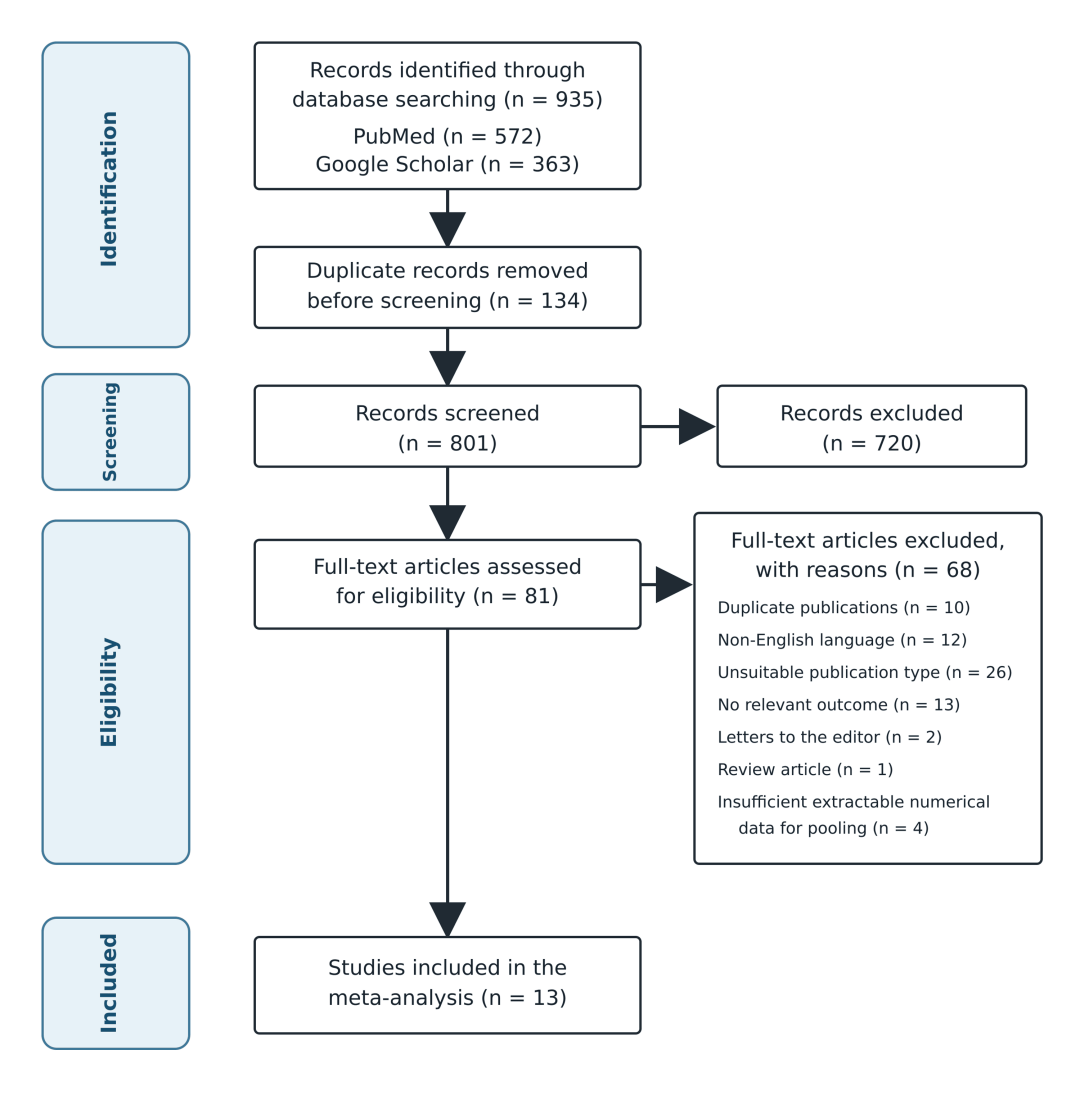
PRISMA flow diagram of the study-selection process. PRISMA: Preferred Reporting Items for Systematic Reviews and Meta-Analyses.

There were 953 articles retrieved from the databases as a result of the queries, 572 extracted from PubMed and 363 from Google Scholar. With the removal of 134 items from duplicate publications, a total of 801 articles remained. Following the examination of the complete texts of the articles for the purpose of screening, 720 articles were excluded. Out of the remaining 81 article, a total of 68 articles were excluded due to concerns with regard to the research subjects; of these, 10 were duplicates, 12 had language other than English, 26 did not have a suitable publication type, 13 did not report a relevant outcome, four did not have sufficient extractable numerical data, two were letters to the editor, and one was a review article. In the end, 13 articles that investigated 17 risk factors were incorporated into the meta-analysis, but only four were compared and studied. Figure 1 illustrates the flowchart of the process that was implemented to locate and assess the articles.

Article Screening

We established the search strategy and terms, reviewed a significant number of papers on the subject of this study, and conducted a preliminary search of electronic databases including PubMEd and Google Scholar, using the following keywords: (Hypertension OR High Blood Pressure) AND (Chronic Kidney Disease OR Chronic Kidney Problems) OR (Hypertension AND Kidney Problems) AND (case-control). Both the PubMed and Google Scholar databases were searched to identify case-control studies published from January 2015 until September 2024. In order to identify references that satisfied the inclusion criteria of our meta-analysis, we conducted numerous searches that combined subject and free text. The search terms employed in English were hypertension, HTN, chronic kidney disease, and CKD. Subsequently, each article was monitored using a search engine to identify further articles that were relevant to the meta-analysis and to obtain the most recent research developments.

Inclusion and Exclusion Criteria

The PICOS principle (patients, intervention, comparison, outcomes, and study design) was used to define the inclusion criteria. The case-control investigation included patients with hypertension (HTN) and chronic kidney disease (CKD) patients without. The literature provides information on CKD, mean age for cases and controls, standard deviation (SD) of ages, cases, and controls numbers, odds ratios, and the 95% confidence interval (CI) of risk factors associated with CKD and HTN. The primary factors studied were renal failure, age, male gender, and smoking, according to the research findings. The literature that has been included in this study is a case-control design. Any study that did not meet the inclusion criteria and/or was earlier than the predetermined range was excluded.

Types and classifications of risk factors

History of diabetes, history of kidney diseases, history of chemotherapy, education level, history of urinary tract infections (UTI), age, male gender, smoking, low protein intake, iron-deficiency anemia, low income, hyperuricemia, and lack of exercise were the main risk factors in the included studies. However, we discovered that renal failure, age, male gender, and smoking were the most prevalent risk factors in the studies we analyzed.

Statistical methods

The statistical analysis was conducted using the Comprehensive Meta-Analysis V4 software (Biostat Inc., Englewood Cliffs, NJ, USA) [11]. The evaluation index was OR. The 95% CI was employed to express each effect. The literature's heterogeneity was assessed using a chi-square-based Q-test. The heterogeneity was deemed to be high if P>0.1 and I250%, and a random-effects model (REM) was employed for the meta-analysis. Furthermore, a study was conducted to investigate the correlation between the research factors associated with each binary variable and dimensionality reduction (DR). The aggregated statistics of multiple studies were considered statistically significant if P≤0.05; conversely, they were not considered statistically significant if P≤0.05.

## Review

Search results and basic document information

The fundamental information and the risk factors for CKD and HTN from the chosen articles are presented in Table 1. The table encompasses the following: the year of publication, the mean, the standard deviation (SD), the number of cases and controls, the risk factors identified in each paper, and the region of publication. The reviewed studies explored a variety of risk factors for HTN and CKD. The commonly identified risk factors included HTN, diabetes, age, smoking, and family history of kidney diseases. In specific regions, such as rural India and Shiraz, Iran, specific factors were highlighted, including low birth weight, a history of chemotherapy, UTI, and a low intake of potassium or protein. Environmental and lifestyle factors also played a significant role, such as exposure to contaminated water, occupational exposure to herbicides, and cadmium, particularly in regions like India, Pakistan, and Thailand. Lifestyle habits including chewing betel, pet ownership, and water treatment were significant in Sri Lanka, while factors like aldosterone excess, kidney size, and hyperuricemia were prominent in Spain and Taiwan. Socioeconomic factors like low education were identified in China, and gender-specific factors like male gender were explored in Taiwan. The research demonstrates that risk variables differ significantly based on regional and environmental contexts, highlighting the complex characteristics of chronic kidney disease and hypertension.

**Table 1:** The basic information from the articles that explored the risk factors for CKD and HTN CKD: Chronic kidney disease; HTN: hypertension; SBP: systolic blood pressure; UTI: urinary tract infection.

Study	Year	Risk Factors	Area
Ghelichi-Ghojogh et al. [11]	2022	Low Birth Weight, History of Diabetes, History of Kidney Diseases, History of Chemotherapy, Education Level, History of UTI, Burns	Shiraz, Iran
Pry et al. [12]	2021	Chewing Betel, Age, Owning a Pet Dog, Water Treatment, Pests in the House	North Central and Uva Province, Sri Lanka
Fernández-Argüeso et al. [13]	2021	Aldosterone Excess, Chronic Kidney Disease, Essential Hypertension	Madrid, Spain
Zhang et al. [14]	2018	Hypertension, High SBP, High Biomarker Levels	Puerto Rico , United States
Peng et al. [15]	2020	Long-Term Hypertension, CKD, High SBP	Taiwan
Shaik and Jain [16]	2021	Hypertension, Kidney size	Indore, India
Evans et al. [17]	2021	Low Potassium Intake, Anemia, Low Protein Intake, Hypertension, Diabetes, Age, Smoking, Use of Contaminated Water, Occupational Exposure to Herbicides	Rural India​
Schneider et al. [18]	2018	Hypertension, CKD	Germany​
Long et al. [19]	2024	H-Type Hypertension, Low Education	Hunan, China
Mubarik et al. [20]	2019	Age, Smoking, Diabetes, Kidney Diseases, Family History of Hypertension	Rawalpindi, Pakistan
Palo et al. [21]	2021	Hypertension, Diabetes, Drinking Contaminated Water	Odisha, India
Yimthiang et al. [22]	2023	Hypertension, Diabetes, Cadmium Exposure	Nakhon Si Thammarat Province, Thailand
Su et al. [23]	2015	Ageing, Anemia, Hypertension, Diabetes, Hyperuricemia, Smoking, Male Gender, Lack of Exercise, Groundwater Use	Taiwan

**Figure 2:**
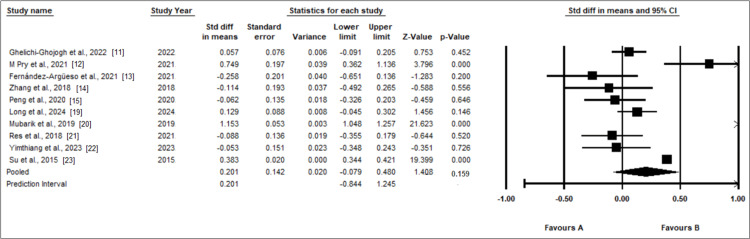
Meta-Analysis Results for Standard Difference in Means in HTN and CKD studies. CKD: Chronic kidney disease; HTN: hypertension.

Table 2 shows the mean, standard deviation (SD), and number of cases and controls in the articles that explored the risk factors for CKD and HTN. The results generated from Comprehensive Meta-Analysis V4 of Table 2 data are shown in Figure 2. The studies explored several risk factors for CKD and HTN. The overall effect size across the studies was small, with a random effects model showing a standardized mean difference (SMD) of 0.201, indicating a positive but not statistically significant association (95% CI: -0.079 to 0.480, Z=1.408, p=0.159). The prediction CI ranged from -0.844 to 1.245, indicating that following studies may reveal a broad spectrum of impacts.

**Table 2:** Mean, Standard deviation (SD), and number for cases and controls in the articles exploring the risk factors for CKD and HTN. CKD: Chronic kidney disease; HTN: hypertension.

Study	Study Year	Mean Age of Cases (years)	SD Ages of Cases	Number of Cases	Mean Age of Controls (years)	SD Age of Controls	Number of Controls
Ghelichi-Ghojogh et al. [11]	2022	59.6	12.4	350	58.9	12.2	350
Pry et al. [12]	2021	57.5	9.6	56	49.5	11.7	54
Fernández-Argüeso et al. [13]	2021	57.7	14.9	50	60.9	9.3	50
Zhang et al. [14]	2018	67	9.0	128	68	8.0	34
Peng et al. [15]	2020	62.4	9.9	256	63.0	8.9	70
Long et al. [19]	2024	65.00	15.56	257	63.00	15.56	257
Mubarik et al. [20]	2019	43.32	9.7	549	31.8	10.1	1451
Palo et al. [21]	2021	49.00	10.62	83	50.00	11.78	153
Yimthiang et al. [22]	2023	59.9	9.7	88	60.4	9.2	88
Su et al. [23]	2015	59.8	15.2	5328	54.0	15.1	5135

Several individual studies showed significant associations. For instance, Mubarik et al. (2019) [20] had a large and highly significant effect (SMD=1.153, 95% CI: 1.048 to 1.257, Z=21.623, p<0.001), indicating a strong positive association between risk factors and CKD or HTN. Similarly, Pry et al. (2021) [12] and Su et al. (2015) [23] showed positive associations with standardized mean differences of 0.749 and 0.383, respectively, both highly significant (p<0.001). On the other hand, other studies reported non-significant results. For instance, Fernández-Argüeso et al. (2021) [13], Zhang et al. (2018) [14], and Palo et al. (2021) [21] all had negative standardized mean differences, though none reached statistical significance (p-values of 0.200, 0.556, and 0.520, respectively). Similarly, studies such as Ghelichi-Ghojogh et al. (2022) [11] and Yimthiang et al. (2023) [22] also reported non-significant results (p=0.452 and p=0.726).

**Figure 3:**
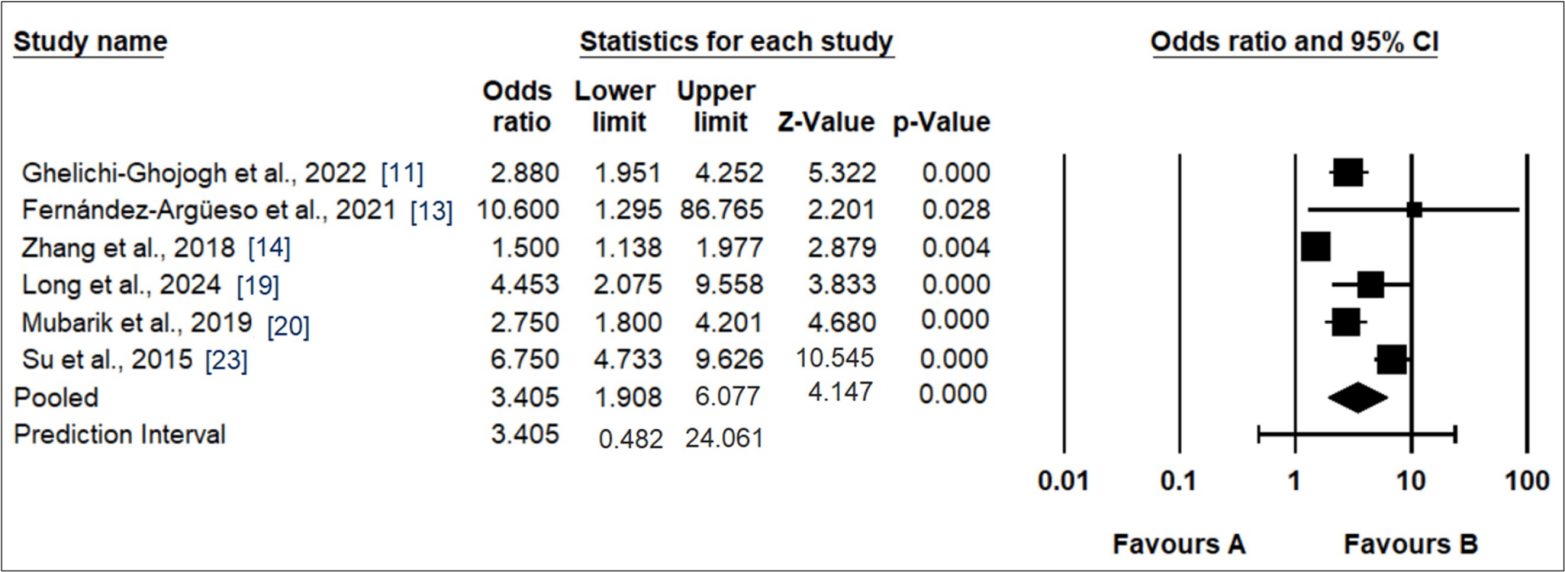
Meta-Analysis Results for Odds Ratio and 95% CI in HTN and CKD studies. CKD: Chronic kidney disease; HTN: hypertension.

Table 3 shows the OR and CI for HTN and CKD, while Figure 3 displays the generated output results. The random-effects model was employed for the analysis, allowing inferences to be made to a broader population beyond the analyzed studies. The mean effect size across the studies was 3.405 (95% CI: 1.908 to 6.077), suggesting a significant association between the risk factors and these conditions. The analysis revealed substantial heterogeneity among the studies, as indicated by the Q-statistic (Q=46.238, five degrees of freedom, p<0.001). This suggests that the true effect sizes differ between studies, and they do not share a common effect size.

**Table 3:** Odds Ratio (OR) and Confidence Interval (CI)  for HTN and CKD CKD: Chronic kidney disease; HTN: hypertension.

Study	Odds Ratio (OR)	Lower 95% Confidence Interval (CI)	Upper 95% Confidence Interval (CI)
Ghelichi-Ghojogh et al. 2022 [11]	2.88	1.95	4.25
Fernández-Argüeso et al. 2021 [13]	10.6	1.3	87.1
Zhang et al. 2018 [14]	1.50	1.14	1.98
Long et al. 2024 [19]	4.453	2.075	9.56
Mubarik et al. 2019 [20]	2.75	1.80	4.2
Su et al. 2015 [23]	6.75	4.76	9.68

The prediction interval, ranging from 0.482 to 24.061, reflects that the true effect size in future comparable studies could vary widely, emphasizing the variability among the studies included in this analysis. Numerous individual investigations indicated notably robust relationships. Su et al. (2015) [23] found an OR of 6.750 (95% CI: 4.733 to 9.626, p<0.001), while Fernández-Argüeso et al. (2021) [13] indicated a significant but broad OR of 10.600 (95% CI: 1.295 to 86.765, p=0.028). Additional research, including Long et al. (2024) [19] and Mubarik et al. (2019) [20], demonstrated significant relationships, with ORs of 4.453 and 2.750, respectively (both p<0.001). Zhang et al. (2018) [14] reported a moderate OR of 1.500 (95% CI: 1.138 to 1.977, p=0.004), which is still statistically significant. The meta-analysis highlights a robust correlation between risk variables and the onset of CKD or HTN, with the majority of studies indicating a significant positive connection. The variability indicated by the prediction interval suggests that additional research may elucidate the magnitude of these connections.

Meta-analysis results of decreased renal function as a risk factor

**Figure 4:**
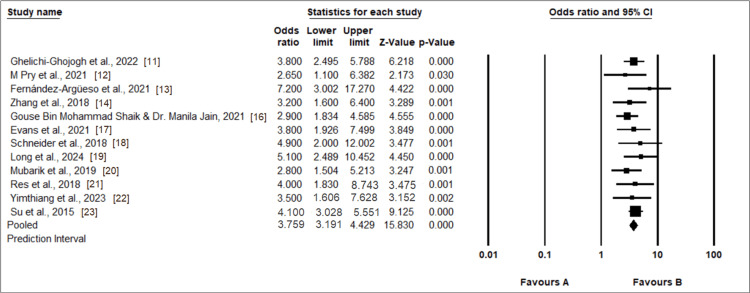
Forest map for HTN and CKD as a risk factor of decreased renal function. CKD: Chronic kidney disease; HTN: hypertension

Table 4 shows the adjusted OR, CI, and p-values for decreased renal function as a risk factor for hypertension and CKD, while Figure 4 shows the generated meta-analysis across 12 studies using a random-effects model. The overall OR was 3.759 (95% CI: 3.191 to 4.429), indicating a significant association between decreased renal function and the development of hypertension or CKD. A Z-value of 15.830 (p<0.001) strongly supports the conclusion that decreased renal function significantly increases the risk of these conditions.

**Table 4:** Adjusted OR, CI, and p-values for Decreased Renal Function as a risk factor for HTN and CKD. CKD: Chronic kidney disease; HTN: hypertension

Study	Adjusted OR	95% CI (Lower)	95% CI (Upper)	P-value
Ghelichi-Ghojogh et al. 2022 [11]	3.80	2.50	5.80	<0.001
Pry et al. 2021 [12]	2.65	1.10	6.38	0.031
Fernández-Argüeso et al. 2021 [13]	7.20	3.00	17.26	<0.001
Zhang et al. 2018 [14]	3.20	1.60	6.40	0.001
Shaik and Jain, 2021 [16]	2.90	1.80	4.50	<0.001
Evans et al. 2021 [17]	3.80	1.90	7.40	0.001
Schneider et al. 2018 [18]	4.90	2.00	12.00	0.001
Long et al. 2024 [19]	5.10	2.50	10.50	<0.001
Mubarik et al. 2019 [20]	2.80	1.50	5.20	0.002
Palo et al. 2021 [21]	4.00	1.80	8.60	0.002
Yimthiang et al. 2023 [22]	3.50	1.60	7.60	0.001
Su et al. 2015 [23]	4.10	3.00	5.50	<0.001

Several studies reported highly significant findings. For example, Su et al. (2015) [23] found an OR of 4.100 (95% CI: 3.028 to 5.551, p<0.001), while Fernández-Argüeso et al. (2021) [13] showed an OR of 7.200 (95% CI: 3.002 to 17.270, p<0.001). Additionally, Ghelichi-Ghojogh et al. (2022) [11] reported an OR of 3.800 (95% CI: 2.495 to 5.788, p<0.001), and Long et al. (2024) [19] had an OR of 5.100 (95% CI: 2.489 to 10.452, p<0.001), all of which emphasize the strong link between decreased renal function and these diseases. The prediction interval for future studies ranged from 0.482 to 24.061, reflecting the potential variability in effect sizes in future research. While decreased renal function was consistently shown to be a significant risk factor for HTN and CKD, the substantial heterogeneity among the studies suggests that the strength of this association may vary depending on specific populations or environmental contexts.

Meta-analysis results of smoking as a risk factor

**Figure 5:**
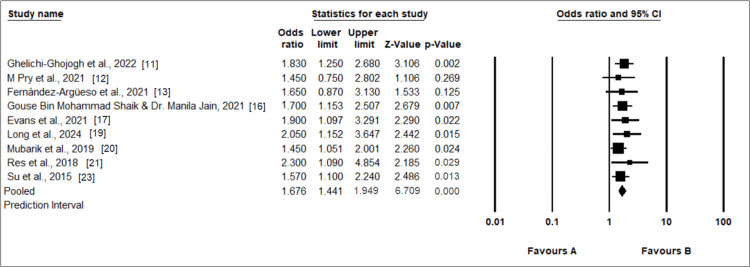
Forest map for HTN and CKD as a risk factor of smoking. CKD: Chronic kidney disease; HTN: hypertension

Table 5 shows the adjusted OR, CI, and p-values for smoking as a risk factor for HTN and CKD, while Figure 5 shows the generated meta-analysis across nine studies using a random-effects model. The overall OR, based on a random-effects model, was 1.676 (95% CI: 1.441 to 1.949, p<0.001), indicating a significant association between smoking and the increased risk of these conditions. Several individual studies showed significant results. For example, Ghelichi-Ghojogh et al. (2022) [11] reported an OR of 1.830 (95% CI: 1.250 to 2.680, p=0.002), and Shaik and Jain (2021) [16] found an OR of 1.700 (95% CI: 1.153 to 2.507, p=0.007), both highlighting a notable link between smoking and elevated risk. Additionally, Long et al. (2024) [19] reported an OR of 2.050 (95% CI: 1.152 to 3.647, p=0.015), and Evans et al. (2021) [17] found an OR of 1.900 (95% CI: 1.097 to 3.291, p=0.022), indicating that smoking significantly contributes to the development of HTN and CKD in these studies.

**Table 5:** Adjusted OR, CI, and p-values for Smoking as a risk factor for HTN and CKD. OR: Odds ratio; CI: confidence interval; CKD: Chronic kidney disease; HTN: hypertension.

Study	Adjusted OR	95% CI (Lower)	95% CI (Upper)	P-value
Ghelichi-Ghojogh et al. 2022 [11]	1.83	1.25	2.68	0.001
Pry et al. 2021 [12]	1.45	0.75	2.80	0.251
Fernández-Argüeso et al. 2021 [13]	1.65	0.87	3.13	0.125
Shaik and Jain, 2021 [16]	1.57	1.10	2.24	0.013
Evans et al. 2021 [17]	1.70	1.15	2.50	0.008
Long et al. 2024 [19]	1.90	1.10	3.30	0.022
Mubarik et al. 2019 [20]	2.05	1.15	3.64	0.012
Palo et al. 2021 [21]	1.45	1.05	2.00	0.024
Su et al. 2015 [23]	2.30	1.10	4.90	0.026

Conversely, some studies, such as Pry et al. (2021) [12] and Fernández-Argüeso et al. (2021) [13], did not reach statistical significance, with ORs of 1.450 (95% CI: 0.750 to 2.802, p=0.269) and 1.650 (95% CI: 0.870 to 3.130, p=0.125), respectively. The overall analysis supports the conclusion that smoking is a significant risk factor for HTN and CKD. However, the variability in effect sizes across studies, as shown by the prediction interval, suggests that the strength of this association may differ depending on the population or study design.

Meta-analysis results of male gender as a risk factor

**Figure 6:**
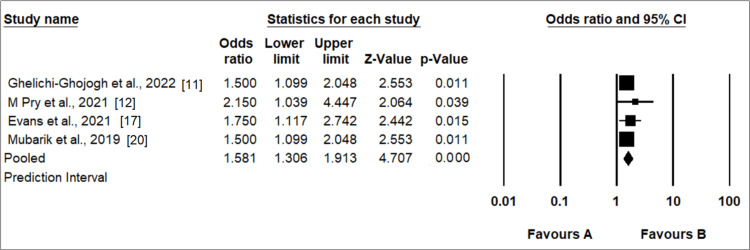
Forest map for HTN and CKD as a risk factor of male gender. HTN: Hypertension; CKD: chronic kidney disease.

Table 6 shows the adjusted OR, CI, and p-values for male gender as a risk factor for HTN and CKD, while Figure 6 shows the generated meta-analysis across four studies using a random-effects model. The analysis indicates that male gender is a significant risk factor for both HTN and CKD. Ghelichi-Gh et al. [11] reported an adjusted odds ratio (OR) of 1.50, meaning that men are 50% more likely to develop these conditions compared to women, with a 95% CI of 1.099 to 2.048 and a p-value of 0.011, confirming statistical significance. Similarly, Pry et al. [12] found an even higher OR of 2.15 (95% CI: 1.039-4.447, p=0.039), indicating more than double the risk for men. Evans et al. reported an OR of 1.75 (95% CI: 1.117-2.742, p=0.015), suggesting a 75% increased risk for men. Mubarik et al. also found an OR of 1.50 (95% CI: 1.099-2.048, p=0.011), identical to Ghelichi-Gh’s results. The random-effects model incorporating these data produced an odds ratio of 1.581 (95% CI: 1.306-1.913, p<0.001), supporting male gender as a major risk factor for hypertension and chronic kidney disease across the examined studies.

**Table 6:** Adjusted OR, CI, and p-values for male gender as a risk factor for HTN and CKD. OR: Odds ratio, CI: confidence interval, HTN: hypertension, CKD: chronic kidney disease.

Study	Adjusted OR	95% CI (Lower)	95% CI (Upper)	P-value
Ghelichi-Ghojogh et al. 2022 [11]	1.50	1.10	2.05	0.012
Pry et al. 2021 [12]	2.15	1.04	4.45	0.037
Evans et al. 2021 [17]	1.50	1.10	2.05	0.012
Mubarik et al. 2019 [20]	1.75	1.12	2.75	0.018

By applying meta-analysis for the risk factors of HTN and CKD, the test results revealed the findings regarding the association as shown in Table 7. The overall OR for age as a risk factor was 1.155 (95% CI: 0.757-1.762), which showed no significant association between age and the risk of HTN and CKD. The results also showed that smoking was associated with an increased risk of HTN and CKD, with an overall OR of 1.732 (95% CI: 1.208-2.482). Heterogeneity for both factors was low (I²=0.0%, p=0.999), indicating consistency among studies, as evidenced by the Cochran's Q test value of 0.2427 and 0.6018, respectively.

**Table 7:** Meta-Analysis of Risk Factors for HTN and CKD: Overall Odds Ratios (95% CI), Heterogeneity Assessment, Cochran’s Q test value HTN: Hypertension; CKD: chronic kidney disease; CI: confidence interval.

Risk Factors	Overall OR (95% CI)	Heterogeneity (I², p-value)	Cochran’s Q
Smoking	1.732 (1.208-2.482)	I²=0.0%, p=0.999	0.6018
Male Gender	1.7013 (1.082-2.674)	I²=0.0%, p=0.938	0.4087
Renal Impairment	3.846 (2.817-5.251)	I²=0.0%, p=0.987	3.2311

The analysis showed that male gender is a significant risk factor, with an OR of 1.7013 (95% CI: 1.082-2.674). Also, no heterogeneity was found (I²=0.0%, p=0.938), with Cochran’s Q test value of 0.4087. Renal impairment showed the highest risk association, with an OR of 3.846 (95% CI: 2.817-5.251), indicating a strong correlation with HTN and CKD. Heterogeneity was minimal (I²=0.0%, p=0.987), and Cochran’s Q test was 3.2311.

Discussion

This meta-analysis identified a strong association between HTN and CKD, with smoking and male sex associated with CKD. These findings should be interpreted as associations rather than evidence that each factor independently causes CKD. Overall, the observed pattern is consistent with contemporary reviews describing HTN and CKD as closely interconnected disorders, with each condition capable of contributing to the development or progression of the other [6,7,9].

The association between HTN and impaired renal function is biologically plausible. Persistent elevation of systemic and intraglomerular pressure can promote endothelial dysfunction, renal arterial and arteriolar remodeling, glomerulosclerosis, albuminuria, and progressive nephron loss. Conversely, declining kidney function can aggravate HTN through sodium retention, activation of the renin-angiotensin-aldosterone and sympathetic nervous systems, and impaired pressure natriuresis [6,7]. This reciprocal relationship explains why HTN is both common in CKD and associated with its progression. However, because many of the included studies were observational, the present analysis cannot establish the temporal direction of this relationship. The findings should therefore be described as supporting a bidirectional association rather than confirming bidirectional causation.

The observed association between smoking and CKD is not uniform across the available literature. In an Iranian case-control study, smoking was associated with CKD in the unadjusted analysis but was not retained among the independently associated factors after multivariable adjustment [11]. Similarly, the Taiwanese multicenter study by Su et al. found no independent association between smoking and CKD after adjustment for accompanying demographic and clinical variables [23]. These differences may reflect variation in smoking definitions, cumulative tobacco exposure, duration of smoking, residual confounding, and the characteristics of the populations examined. Accordingly, the present pooled finding supports smoking as a modifiable factor associated with CKD but does not independently establish a causal relationship. Nevertheless, smoking cessation remains clinically important because of its established cardiovascular benefits and the substantial cardiovascular burden affecting patients with both HTN and CKD.

The association with male sex is consistent with the findings of Su et al., who identified male sex and ageing as factors associated with CKD across different comorbidity groups [23]. Schneider et al. also found that younger age and female sex were associated with better blood pressure control among patients with CKD, which may partly contribute to the differences observed between demographic groups [18]. The effect of age is unlikely to be explained by cumulative exposure to HTN alone. Age-related nephron loss, vascular stiffening, diabetes, medication exposure, and increasing multimorbidity may also contribute. Furthermore, age and sex are components of commonly used creatinine-based estimated glomerular filtration rate equations, meaning that part of their association with eGFR-defined CKD may be influenced by the method used to classify renal function. These findings therefore require cautious interpretation. The term “sex” should also be used instead of “gender” unless gender identity was specifically measured.

The geographic diversity of the included studies increases the applicability of the findings but probably contributed to the observed heterogeneity. Studies conducted in Iran, Sri Lanka, rural India, and Taiwan have reported different patterns of clinical, behavioral, and environmental factors associated with CKD and HTN [11,12,17,21,23]. For example, Palo et al. found that having HTN for more than five years was strongly associated with CKD in Odisha, India, while drinking-water source, locally produced alcohol, and dietary characteristics were also associated with CKD [21].

In contrast, a pilot study from Sri Lanka did not identify occupational pesticide exposure or alcohol consumption as significant factors associated with CKD of unknown etiology [12]. These differences illustrate how population characteristics, environmental exposures, healthcare access, and study methodology can influence reported associations. Geographic variation should therefore not be attributed solely to lifestyle or environmental factors unless supported by prespecified subgroup analysis or meta-regression.

Differences in CKD definitions and assessment methods may have produced additional heterogeneity. The included studies may have varied in their use of serum creatinine, estimated glomerular filtration rate (eGFR) equations, albuminuria, and the requirement for persistent abnormalities. Studies based on a single renal measurement may misclassify transient reductions in kidney function as CKD. Differences in HTN definitions, blood pressure measurement techniques, treatment status, and the duration and degree of blood pressure control may also influence the estimated associations. These methodological differences should be considered when interpreting the pooled results and their generalizability.

The findings have important clinical implications. Patients with HTN should undergo appropriate assessment for CKD, including measurement of serum creatinine-based eGFR and urinary albumin excretion when available. Blood pressure management remains an essential component of reducing cardiorenal risk [5,7]. However, achieving adequate control can be difficult. In the German CKD cohort, awareness and treatment of HTN were high, but only approximately half of the affected patients achieved the defined blood pressure target [18]. Furthermore, changes in eGFR following intensive blood pressure reduction do not necessarily represent structural kidney injury. Zhang et al. found that incident CKD during intensive systolic blood pressure lowering was accompanied by reductions rather than increases in several kidney-injury biomarkers, suggesting that some early changes may reflect altered renal hemodynamics [14]. Renal function should therefore be monitored carefully, while small early changes in eGFR should be interpreted within the overall clinical context.

Several limitations should be considered. First, most of the included evidence was observational and therefore susceptible to residual confounding and reverse causation. Diabetes, obesity, cardiovascular disease, socioeconomic status, duration and severity of HTN, antihypertensive treatment, and baseline albuminuria may not have been measured or adjusted for consistently. Second, variation in study populations, exposure definitions, and CKD diagnostic methods contributed to heterogeneity that a random-effects model can accommodate but cannot fully explain. Third, self-reported smoking is vulnerable to recall and social-desirability bias, while inconsistent measurement of smoking exposure may obscure dose-response relationships. Fourth, the use of different eGFR equations may have produced differential CKD classification across age and sex groups. Fifth, when CKD is common, odds ratios may be larger than corresponding risk ratios and should not be described as direct measures of risk. Finally, study-level meta-analysis cannot adequately examine interactions among age, sex, smoking, blood pressure control, and accompanying comorbidities, and publication or small-study bias cannot be excluded.

Overall, the findings support targeted CKD detection and comprehensive risk-factor management among individuals with HTN, while emphasizing that the pooled estimates represent associations rather than definitive causal effects. Future studies should use prospective designs, standardized definitions of HTN and CKD, repeated measurements of eGFR and albuminuria, quantitative assessment of tobacco exposure, and consistent adjustment for major metabolic and cardiovascular confounders.

Individual-participant data meta-analysis would be particularly useful for evaluating whether the observed associations differ according to age, sex, CKD stage, blood pressure control, and geographic region.

## Conclusions

This meta-analysis demonstrates the significant correlation between HTN and CKD, highlighting the serious impact these diseases impose on global health. Our findings indicate that impaired renal function, male sex, and tobacco use are significant risk variables that increase the likelihood of CKD in hypertensive populations. These insights underscore the need for efficient HTN management, particularly in regions with limited healthcare access where both conditions are prevalent and often undiagnosed. Considering the strong influence of HTN on renal health, enhanced efforts are essential to promote early detection and management of elevated blood pressure to prevent CKD progression. Public health strategies must prioritize reducing modifiable risk factors, such as smoking and unhealthy lifestyles, while also addressing gender and age-related disparities in disease outcomes.

Future research is required to investigate the fundamental mechanisms of HTN-induced renal impairment, emphasizing the enhancement of diagnostic instruments to accommodate regional and population-specific disparities. Enhancing preventative measures, especially in marginalized areas, will be essential in alleviating the combined burden of HTN and CKD globally.
